# Biohybrids: Textile fibres provide scaffolds and highways for microbial translocation

**DOI:** 10.3389/fbioe.2023.1188965

**Published:** 2023-06-13

**Authors:** Angela Sherry, Bruna Martins Dell’Agnese, Jane Scott

**Affiliations:** ^1^ Hub for Biotechnology in the Built Environment, Department of Applied Sciences, Northumbria University, Newcastle upon Tyne, United Kingdom; ^2^ Hub for Biotechnology in the Built Environment, School of Architecture Planning and Landscape, Newcastle University, Newcastle upon Tyne, United Kingdom

**Keywords:** living materials, microbial translocation, fungal highways, fibre highways, biohybrids

## Abstract

**Introduction:** Living materials (biohybrids, textile-microbial hybrids, hybrid living materials) have gained much attention in recent years with enormous potential for applications in biomedical science, the built environment, construction and architecture, drug delivery and as environmental biosensors. Living materials contain matrices which incorporate microorganisms or biomolecules as the bioactive components. A cross-disciplinary approach, operating at the intersection of creative practice and scientific research, incorporated textile technology and microbiology to demonstrate textile fibres providing microbial scaffolds and highways during this study.

**Methods:** The study evolved from previous research which showed bacteria utilising the water layer surrounding fungal mycelium for motility, termed the ‘fungal highway’, which led to the investigation of the directional dispersal of microbes across a range of fibre types (natural and synthetic). The application of the study centred around the potential for biohybrids to be used as a biotechnology to improve oil bioremediation through seeding of hydrocarbon-degrading microbes into polluted environments via fungal or fibre highways, therefore treatments in the presence of crude oil were tested. Furthermore, from a design perspective, textiles have huge potential to act as a conduit for water and nutrients, essential to sustain microorganisms within living materials. Using the moisture absorption properties of natural fibres, the research explored how to engineer variable liquid absorption rates using cellulosics and wool to produce shape-changing knitted fabrics suitable for adaptation to oil spill capture.

**Results:** At a cellular scale, confocal microscopy provided evidence to show that bacteria were able to utilise a water layer surrounding the fibres, supporting the hypothesis that fibres can aid bacterial translocation through their use as ‘fibre highways’. A motile bacterial culture, *Pseudomonas putida*, was shown to translocate around a liquid layer surrounding polyester, nylon, and linen fibres, yet no evidence of translocation was apparent on silk or wool fibres, suggesting microbes elicit different responses to specific fibre types. Findings showed that translocation activity around highways did not diminish in the presence of crude oil, known to contain an abundance of toxic compounds, in comparison to oil-free controls. A design series demonstrated the growth of fungal mycelium (*Pleurotus ostreatus*) through knitted structures, highlighting the ability for natural fabrics to provide a scaffold to support microbial communities whilst retaining the ability to undergo environmentally responsive shape-change. A final prototype, Ebb&Flow, demonstrated the potential to scale up the responsive capacities of the material system using locally produced UK wool. The prototype conceptualised both the uptake of a hydrocarbon pollutant by fibres, and the translocation of microbes along fibre highways.

**Discussion:** The research works towards facilitating the translation of fundamental science and design into biotechnological solutions that can be used in real world applications.

## 1 Introduction

Living materials contain matrices which incorporate microorganisms or biomolecules as the bioactive components. Such living materials (biohybrids, hybrid living materials (HLM)) and engineered living materials (ELM, reviewed in Gilbert & Ellis, 2019; Nguyen et al., 2018; Rodrigo-Navarro et al., 2021) have gained much attention in recent years with enormous potential for applications in biomedical science, the built environment, construction and architecture, drug delivery and as environmental biosensors. Recent advances include programmable (Gilbert et al., 2021) and stimuli-responsive (reviewed in Rivera-Tarazona et al., 2021) engineered living materials. As demand continues to increase, these new generation materials will likely replace the more traditional carbon-intensive production of materials across a number of markets, including construction, architecture and fashion. Living materials can offer low energy circular models of material biofabrication operating at ambient temperature without toxic by-products (Gilbert & Ellis., 2019).

Textiles are unique in their ability to transform materials across multiple scales, from individual fibres into hyper-specified fabrics tailored for tensile systems or to act as reinforcement for compressive loads. Furthermore, textile materials have the potential to provide highly functionalised surfaces with the potential to tailor structure and function through combinations of fibres, yarns and fabric construction processes for specific applications. Textiles are lightweight, and can be manufactured from natural, synthetic or biosynthesised materials using circular production models. These and other physicochemical properties of textiles (e.g., fibre morphology, wettability, hydrophilicity/hydrophobicity) provided scope to incorporate microbiology methodologies with textile design and technology in order to explore microbe:textile (biohybrid) relationships and fabricate novel types of materials incorporating textile processes for a range of different purposes and applications (e.g., Helberg et al., 2019).

The movement of bacterial communities along fungal mycelium has previously been investigated and termed the ‘fungal highway’ (Kohlmeier et al., 2005; Furuno et al., 2010; Frey-Klett et al., 2011; Pion et al., 2013; Zhang et al., 2018). A liquid film surrounds the fungal mycelium in which microorganisms can translocate towards a chemical with the mycelium acting as a vector. Previous studies have shown the mobilisation and dispersal of polycyclic aromatic hydrocarbon-degrading bacteria on fungal mycelium and reported that fungal highways have the ability to improve the bioavailability of pollutants in environments, such as soils (Kohlmeier et al., 2005; Furuno et al., 2010). Herein, we utilised existing knowledge and the body of evidence related to fungal highways to investigate interactions and the translocation of microbes on a range of fibre types (natural and synthetic), termed *fibre highways*.

There is a global legacy of hydrocarbon contaminated ecosystems where options for novel or more efficient remediation strategies should continue to be a research focus (Daghio et al., 2017; Sherry et al., 2017; Mejeha et al., 2019; Sherry et al., 2020). Despite a range of potential applications, this research centred around the potential to use living materials to improve crude oil bioremediation through seeding hydrocarbon-degrading microbes into polluted environments via microbe:textile biohybrids.

## 2 Materials and methods

Growth experiments and microscopy visualisation were used to determine the translocation of bacterial cells on fungal mycelium or textile fibres in relation to hydrocarbon biodegradation. While knitted fabrics were designed to investigate ways to apply fibre highways at a fabric scale through investigation of liquid absorption and retention related to responsive behaviours, and the potential for knit to act as a scaffold for mycelium growth.

### 2.1 Textile fibres

Merino wool (www.wools.co.uk), silk, polyester (www.hobbycraft.co.uk), natural linen flax (George Weil, www.fibrecrafts.com) and nylon (monofilament Fladen Vantage, www.empressmills.co.uk) were sourced as a range of natural and synthetic fibres on which to test the translocation ability of a motile bacterial culture of *Pseudomonas putida* (Section 2.3.2). Fibres were raw (with no finish) and were selected for either high levels of bacterial adhesion, for example, polyester and nylon (Møllebjerg et al., 2021) or fibres characterised as ‘antibacterial’ such as flax, wool and silk (Zimniewska & Goślińska-Kuźniarek., 2016; Caven et al., 2019; Varshney et al., 2021, respectively). In addition, fibres were selected suitable for applications in programmable knitting (Scott., 2018) to support the design investigation (Section 2.4).

#### 2.1.1 Light microscopy to determine morphology of textile fibres

Light microscopy (Olympus CX21) was used to assess the morphological characteristics, surface features and diameter (µm) of each fibre type.

#### 2.1.2 Physicochemical properties of textiles

Physicochemical properties of the textile fibres were researched and recorded including chemical composition, static electrical charge, hydrophobicity/hydrophilicity, moisture regain (%), tenacity (N/tex), and specific gravity (g cm^-3^) (Gohl & Vilensky, 1983; ASTM., 2004; Yurtseven., 2004; Bunsell., 2018; Laitala et al., 2018).

#### 2.1.3 Wicking tests

At the fabric scale, vertical wicking tests were performed to determine the absorbency ability of wool, silk, polyester, linen and nylon. Wicking tests were conducted according to the method of Hasan et al., 2019 on knitted fabrics (1.61 cm ± 0.03 W × 20.50 cm ± 0.49 H). Initially, fabrics were placed in a drying cabinet (∼50°C) for 24 h and weighed prior to vertical placement in an acrylic wicking rig with reservoir. Distilled water plus colourant (Dr. Ph. Martin’s) was added to the reservoir and each fabric was immersed (to 1 cm depth). The displacement height was measured (every cm) during a total time of 60 min and the fabric was weighed again after wicking. Average velocity (cm s^-1^) was determined from displacement over time on all fabrics tested, as well as final wicking height (cm) and fabric wicking percentage (%) determined from (weight of fabric after wicking (g)—weight of fabric before wicking (g))/weight of fabric after wicking (g) * 100.

### 2.2 Cultivation of bacterial and fungal strains

#### 2.2.1 Pseudomonas putida A3.12

Pseudomonas putida A3.12 (NCIMB 9494), a Gram-negative bacteria isolated from soil (Stanier., 1947) was selected due to its motility and ability to metabolise hydrocarbons. The lyophilised strain was revived in nutrient broth (Oxoid CM3), pH 7.3, 25°C on a gyratory shaker (50 rpm, Multitron INFORS HT, Surrey, United Kingdom) and on nutrient agar, 25°C in a static incubator (Binder, Germany). Single colonies were transferred to fresh liquid nutrient media and grown overnight at 25°C. Bacterial cells used for translocation experiments on fungal mycelium or textile fibres were grown overnight (∼17 h) to a concentration containing 7 × 10^5^–1 × 10^6^ cells mL^-1^.

#### 2.2.2 Cunninghamella elegans Lendner

Cunninghamella elegans Lendner (DSMZ 8217), a fungus originally isolated from estuarine mud, was selected due to its ability to metabolise crude oil (Cerniglia & Perry., 1974). *C. elegans* was cultivated on solid potato dextrose agar (PDA, Scientific Laboratory Supplies Ltd.) at 25°C in a static incubator (Binder, Germany). Periodically, transfers of *C. elegans* to fresh PDA medium was performed aseptically using the tip of a pipette (10 µL).

#### 2.2.3 Pleurotus ostreatus

Pleurotus ostreatus (blue grey oyster mushroom) was available from mushroom box (www.mushroombox.co.uk). Mycelium was supplied as spawn on rye and millet and revived on damp kitchen paper in Petri dishes for 7 days at room temperature (17.4°C ± 0.5) prior to inoculation of knitted textiles (Section 2.4.2).

### 2.3 Bacterial translocation experiments

**FIGURE 1:**
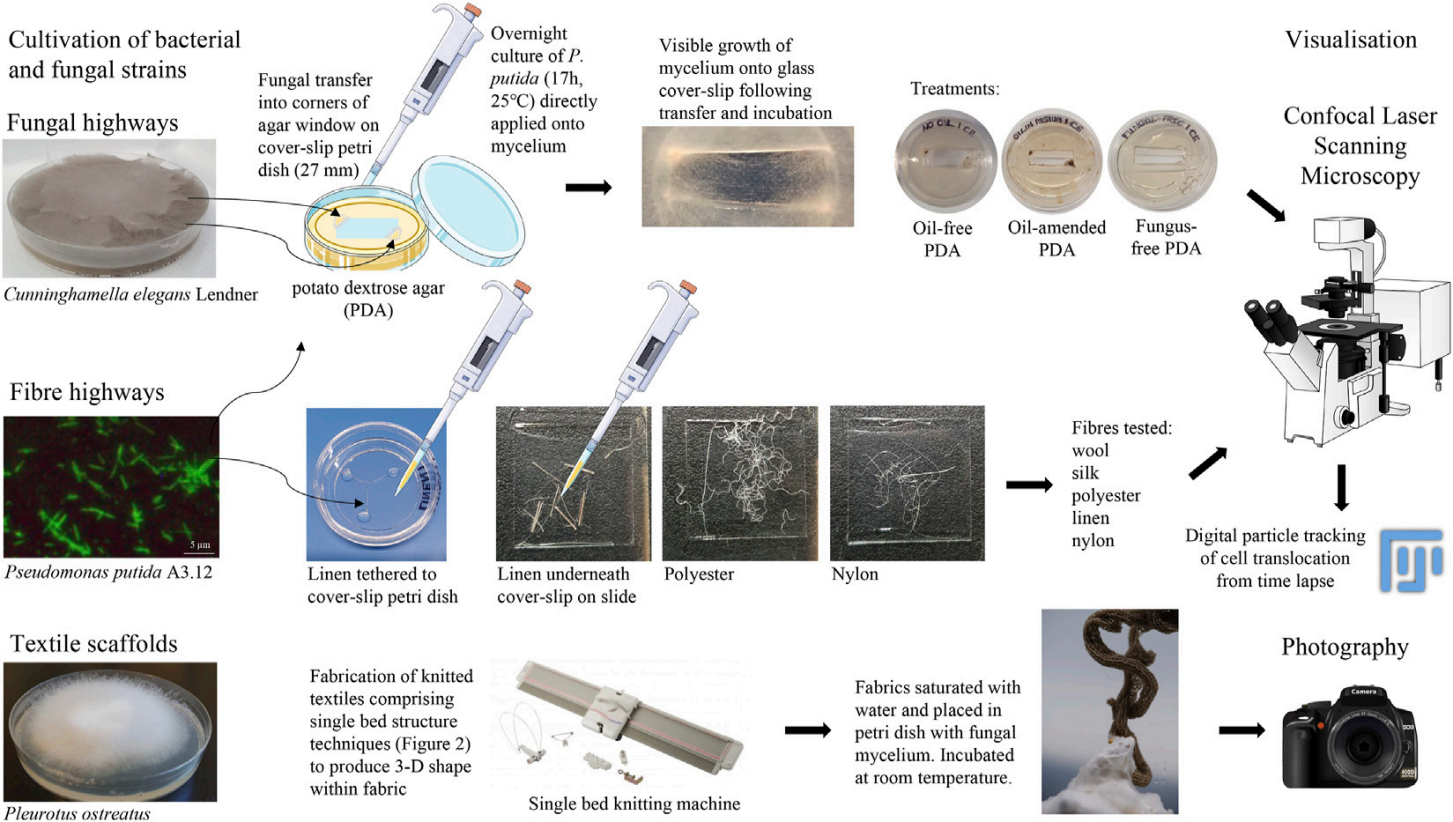
Methodologies of fungal highways, fibre highways and textile scaffolds. Image attributions are included in Supplementary Materials.

Bacterial translocation of *P. putida* on fungal mycelium and textile fibres was assessed (Figure 1), according to:

#### 2.3.1 Translocation on fungal mycelium

PDA (2 mL) was poured into glass cover slip Petri dishes (NUNC 27 mm, Life Technologies Ltd.) and allowed to set. A rectangular pane of PDA was aseptically removed from the centre of the Petri dish and discarded; the resulting corners of the agar were inoculated with *C. elegans* performed aseptically using the tip of a pipette (10 µL)*.* This ensured nutrient provision for the fungal transfers whilst also encouraging growth of the mycelium onto the glass surface to facilitate visualisation of bacterial translocation. *C. elegans* and *P. putida* were grown overnight (∼17 h, 25°C), with *P. putida* cells (20 µL) directly pipetted onto *C. elegans* mycelium immediately prior to microscopy (Figure 1). Experiments without *C. elegans* served as controls to show bacterial movement in the absence of fungal mycelium.

The application of this study centred around the potential use of biohybrids as a biotechnology to enhance oil bioremediation. To assess the activity of *P. putida* translocating on fungal highways in the presence of oil, preliminary tests were prepared with the addition of a North Sea crude oil (10 µL) applied to the surface of the PDA (Figure 1). The crude mix was a Type II kerogen sourced oil with API gravity of ∼38°, and undegraded according to the Peters and Moldowan scale of biodegradation (PM 0; Peters & Moldowan, 1993). Confocal time-lapse images (Section 2.3.3) permitted a comparative analysis of movement of microbes along fungal mycelium in the presence and absence of crude oil amendment.

#### 2.3.2 Translocation on textile fibres

Raw fibres with no finish (wool, polyester, silk, nylon and linen) were autoclaved (20 min, 121°C) and dried overnight (50°C) before aseptic transfer to microscopy slides or glass cover slip Petri dishes where fibres were tethered with PDA to facilitate the microscopy. Addition of *P. putida* cells (20 µL) was performed immediately prior to microscopy (Figure 1).

#### 2.3.3 Visualisation of translocation using Confocal laser scanning microscopy (CLSM)

Confocal laser scanning microscopy (DMi8, Leica Microsystems) was used to visualise the movement of *P. putida* around the mycelium of *C. elegans* and around a range of natural and synthetic fibres (wool, silk, polyester, linen flax, nylon). Bright-field microscopy images were taken using the wide-field image acquisition function (x20, x40 and x63 oil-immersion magnification). Time-lapse images permitted a comparative analysis of movement of microbes along fungal mycelium and textile fibres.

##### 2.3.3.1 Particle tracking of cell translocation

Particle tracking of translocating cells were determined manually in CLSM time-lapse using the MTrackJ plugin in Fiji (Meijering et al., 2012; Schindelin et al., 2012, respectively). The path of single cells were followed by tracking the focal plane and recording x and y movements optically. Time-lapse image properties were 145 × 108 microns (1,392 × 1,040 pixels), 8-bit, grayscale, run time of 30 s consisting of 43 frames at 5 frames per second (fps), therefore 8.6 s video footage length (Supplementary Videos S1A–C to S7A–C, with examples of particle tracking of *P. putida* cells translocating alone, on fungal mycelium or on linen fibre in Supplementary Video S8A–C, respectively). Tracking of 10 individual cells per image in triplicate time lapse were analysed to determine average translocation length (µm, *n* = 30) of particle tracks in a comparative analysis between fibre types and in comparison to cell translocation on fungal mycelium. Translocation of motile *P. putida* cells without the presence of fibres or fungal mycelium were included as a control. Statistical comparisons of translocation length (µm) were performed in pairwise comparisons across all treatments using the non-parametric, independent-samples Kruskal-Wallis test to determine group mean differences (IBM SPSS Statistics, v28).

### 2.4 Design perspective—Knitted fabric Design

Concurrent to scientific experiments, knitted fabrics were developed to investigate ways to apply fibre highways at a fabric scale. Two approaches were adopted. The *Fibre Highways Collection* investigated how different liquid absorption and retention rates observed at a fibre level could translate into responsive behaviours in fabrics, and *knitted scaffolds* explored the potential for knit to act as a scaffold for mycelium growth.

#### 2.4.1 Fabric specifications

**FIGURE 2:**
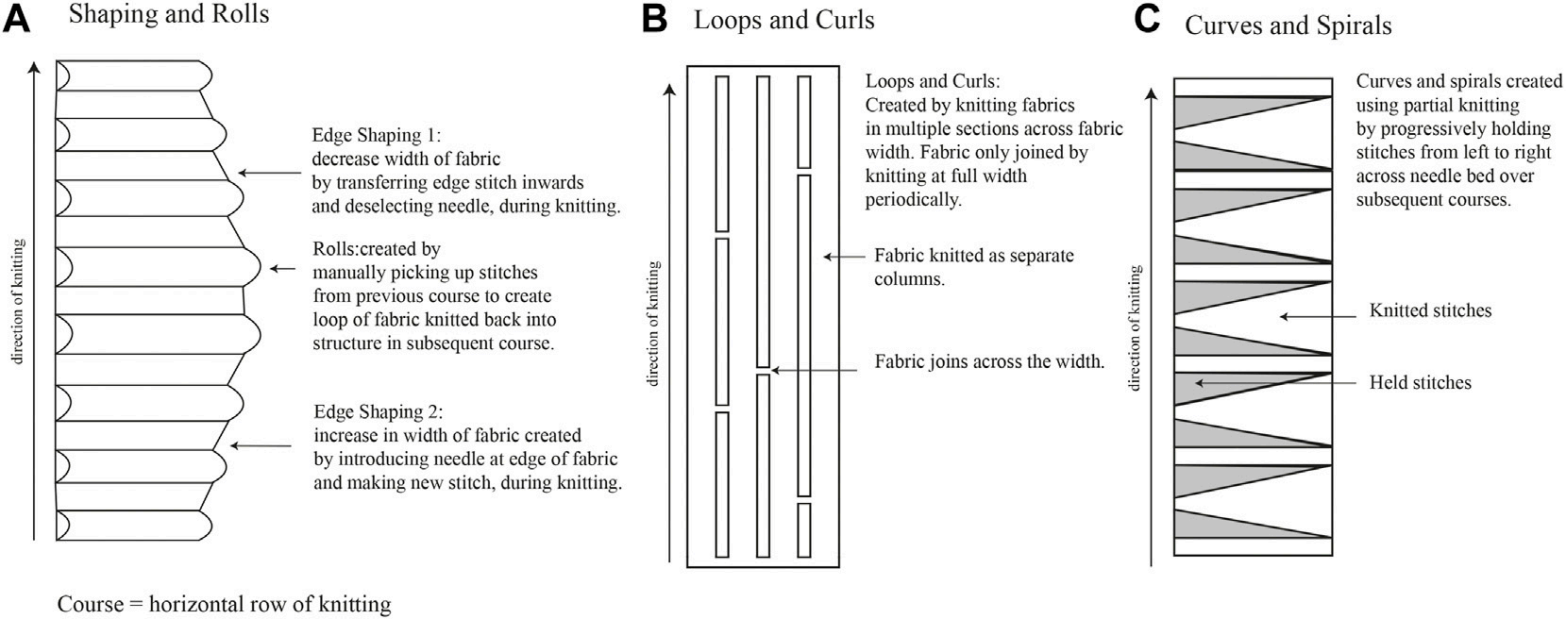
Knitting techniques used for *Fibre Highways Collection*, including shaping and rolls **(A)**, loops and curls **(B)**, and curves and spirals **(C)**.

A range of fabrics were produced on a 7gg single bed machine (Silver Reed, United Kingdom) using 1/24 nm linen (Safilin, France) and 2/28 nm merino wool (www.wools.co.uk). Specific knit techniques comprised shaping and rolls, loops and curls, and curves and spirals (Figures 2A–C, respectively).

#### 2.4.2 Fibre Highways Collection

Fibre highways collection was produced using the shaping and rolls technique (Figure 2A), with sections of edge shaping knitted in 2 ends 1/24 nm linen, and rolls knitted in 2/28 nm merino wool. During knitting the rolls were filled with a selection of raw wool fibres (www.worldofwool.co.uk) to create soft tubular ridges running perpendicular to fabric length.

#### 2.4.3 Fungal inoculation of knitted textiles

Knitted textiles were fabricated from 2 ends 1/24 nm linen (Safilin, France). Knit architecture comprised single bed (plain) structure using techniques to produce 3D shape within the fabric (Figures 2B, C). Fabric was saturated with water and one end of fabric placed in Petri dish with *P. ostreatus* mycelium. Mycelium/knit samples were grown in covered plastic box to prevent light and retain moisture. Growth of mycelium on knitted textiles was photographed (Fujifilm XT-30 with XF80 mm macro lens) regularly over 6 weeks (Figure 1).

## 3 Results

### 3.1 Textile fibre morphology

**FIGURE 3:**
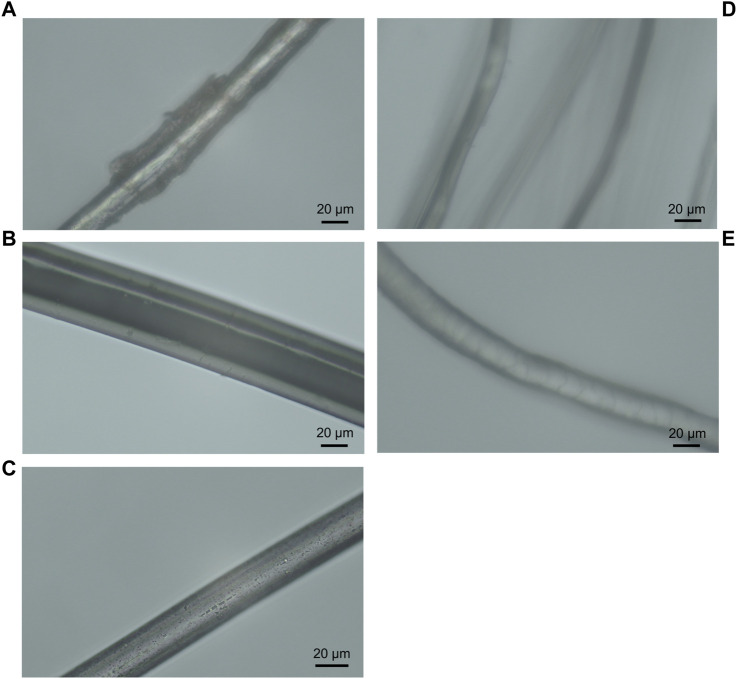
Light microscopy images of Linen flax **(A)**, Nylon **(B)**, Polyester **(C)**, Silk **(D)** and Merino wool **(E)** under 40x objective lens.

To determine whether fibre morphology could possibly affect bacterial translocation, light microscopy was initially used to assess the morphological characteristics and surface features of each fibre type. Surface features included striations and nodes on the stem of linen flax fibres (Figure 3A) and irregular overlapping scales on Merino wool fibres (Figure 3E), in comparison to the smoother fibres of nylon, polyester and silk (Figures 3B–D). Fibre diameter varied among synthetic and natural fibres. Nylon and polyester showed greater diameter thickness (46.3 ± 0.05 and 24.8 ± 0.08 µm, respectively) compared to natural linen, wool and silk (Figure 3; Table 1). Among the natural fibres, linen presented the greater diameter (20.6 ± 0.09, Table 1). Variation in fibre diameter was observed along the length of linen flax and wool fibres (Figures 3A, E; Table 1), whereas diameter was more constant for nylon, polyester and silk (Figures 3B–D; Table 1).

**TABLE 1:** Physicochemical properties and wicking velocities of textile fibres.

Fibre	^a^Chemical composition	^a^Static electrical charge	^a^Hydrophobicity/hydrophilicity	^a^Moisture regain (%)	^a^Tenacity (N/tex)	^a^Specific gravity (g cm^-3^)	^b^Fibre diameter (µm)	^b^Wicking velocity (cm s^-1^)	^b^Height after wicking (cm)	^b^Wicking percentage (%)
Linen	Cellulose	Negative	Hydrophilic	9 to 12	0.4–0.6	1.5	20.6 ± 0.09	0.0019	10.67 ± 0.17	41.49 ± 0.50
Nylon	Polyamides	Positive	Hydrophilic	4.5	0.3–0.9	1.1	46.3 ± 0.05	0.0011	5.33 ± 0.17	52.44 ± 0.61
Polyester	Polyethylene terephthalate	Negative	Hydrophobic	0.4	0.3–0.9	1.4	24.8 ± 0.08	0.0029	13.67 ± 0.33	33.65 ± 1.54
Silk	Fibroin	Negative	Hydrophilic	11	0.2–0.5	1.3	11.9 ± 0.08	0.0000	1.07 ± 0.07	14.14 ± 1.66
Wool	Keratin	Positive	Hydrophilic	13 to 16	0.1–0.2	1.3	14.1 ± 0.14	0.0010	13.33 ± 0.88	53.43 ± 0.84

References:

^a^ Gohl & Vilensky, 1983; ASTM., 2004; Yurtseven., 2004; Bunsell., 2018; Laitala et al., 2018.

^b^ this study.

### 3.2 Physicochemical properties and wicking capacity of textiles

Other physicochemical properties of the textile fibres were recorded including chemical composition, static electrical charge, hydrophobicity/hydrophilicity, moisture regain, tenacity, and specific gravity (Table 1). Wicking velocity was fastest in polyester and linen fabrics (0.0029 and 0.0019 cm s^-1^ respectively), followed by nylon (0.0011 cm s^-1^) (Table 1). Wool showed the slowest velocity to absorb (0.001 cm s^-1^) and silk absorbed the smallest volume of liquid with no visible wicking capacity (Table 1). Wool and nylon showed the capacity to retain the highest percentage of liquid (∼53%) following the time period, in comparison to linen, polyester and silk (Table 1).

### 3.3 Translocation of *Pseudomonas putida* on fungal highways

**FIGURE 4:**
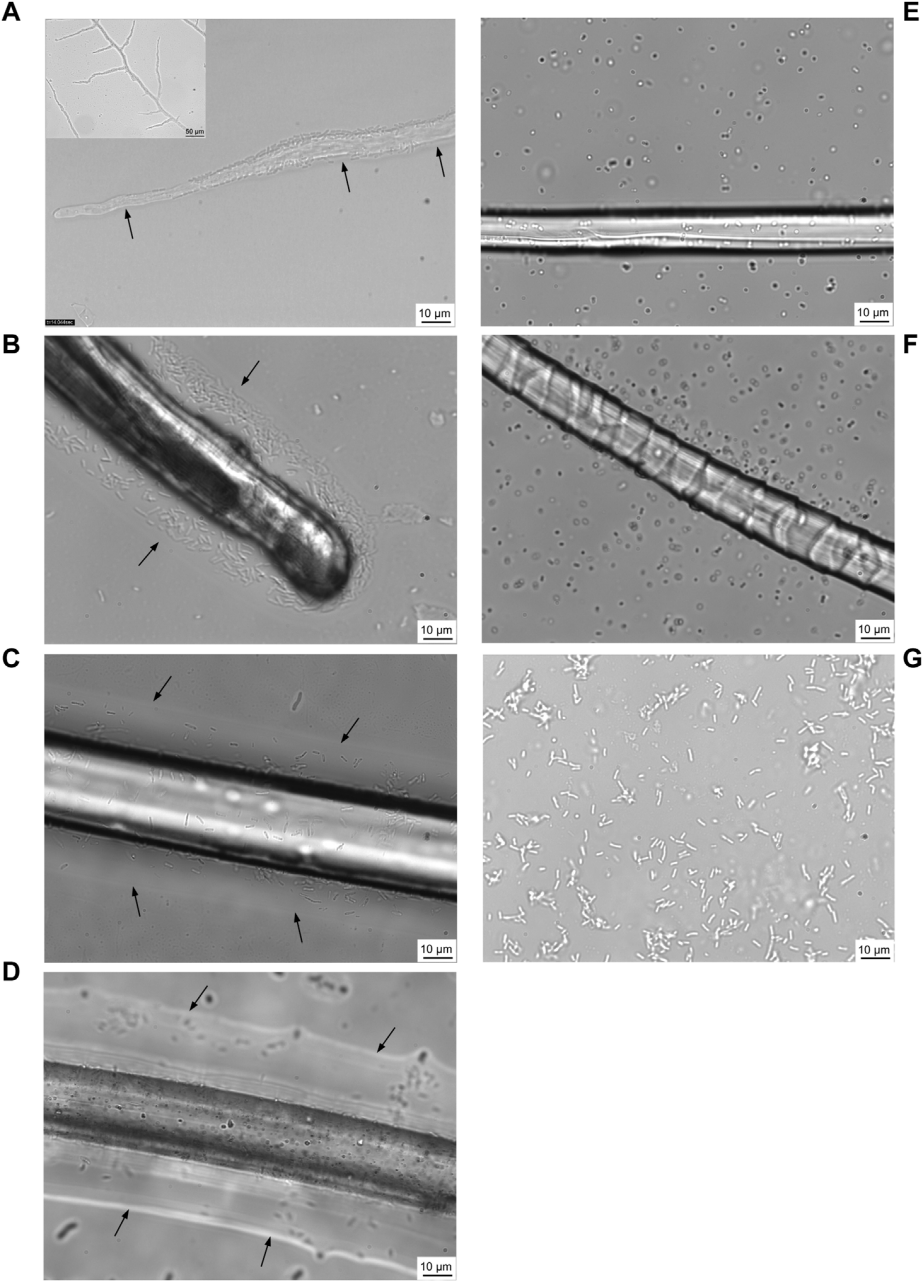
Confocal laser scanning micrographs of motile bacteria, *Pseudomonas putida*, utilising a water layer surrounding the fungal mycelium of *Cunninghamella elegans* for translocation **(A)** (recreated from https://youtu.be/AnsYh6511Ic, Berthold & Wiedling., 2016). Arrows and white shadow surrounding mycelium (inset) indicate water layer. Using a similar approach, five fibres were tested for their ability to support *P. putida* translocation: linen **(B)**, nylon **(C)**, polyester **(D)**, silk **(E)** and wool **(F)**. *P. putida* control without fungal mycelium or fibre present as comparator **(G)**. Confocal time-lapse images are included in (Supplementary Videos S1A–D–S7A–C, respectively).

Translocation of motile *P. putida* cells around the water layer of fungal mycelium from *C. elegans* was observed (Figure 4A and Supplementary Videos 1A-C). This was previously termed the ‘fungal highway’ (Kohlmeier et al., 2005; Berthold & Wiedling., 2016), which was recreated during this research study in order to assist in developing methodologies for testing of bacterial translocation around textile fibres. Furthermore, findings showed that translocation activity around *C. elegans* fungal highways did not diminish in the presence of crude oil which was added to the medium (Supplementary material Video S1D) in comparison to oil-free controls (Supplementary Video S1A–C).

### 3.4 Translocation of *Pseudomonas putida* on fibre highways

It was observed that *P. putida* were indeed able to utilise a water layer surrounding textile fibres in order to translocate (Figures 4B–E). The motile bacterial culture was shown to translocate around a liquid layer surrounding linen, nylon and polyester (Figures 4B–D and Supplementary Videos S2A–C, S3A–C, S4A–C), yet there was little evidence of translocation on a water layer surrounding silk or wool fibres (Figures 4E, F and Supplementary Videos S5A–C, S6–C).

**FIGURE 5:**
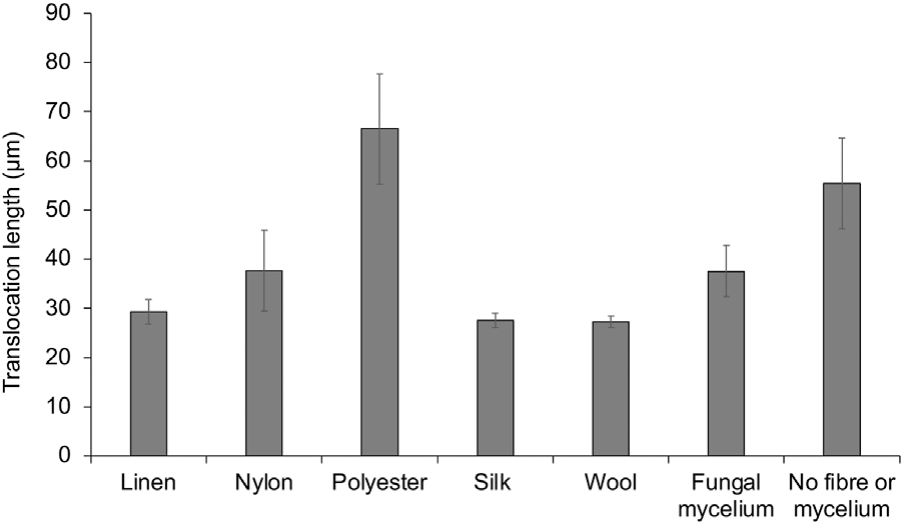
Digital particle tracking of cell translocation length (μm) on fungal mycelium and textile fibre highways.

From confocal time-lapse images, digital particle tracking of cells alone (Supplementary Videos 7A–C) and cells translocating on fungal mycelium and fibres, respectively, was performed (Supplementary Videos S8A–C). *P. putida* cells alone exhibited movement in both the *x* and *y* direction (Figures 4G and Supplementary Video 8A) over the range of x: 4.3—144.5 microns and y: 0.49—108.0 microns. A comparison of the average length (µm) of cells translocating in a water layer surrounding textile fibres and fungal mycelium was determined, in relation to translocation of *P. putida* cells alone (Figure 5). *P. putida* cells translocated significantly longer distances in the water layer surrounding polyester fibres in comparison to the length on linen, nylon, silk and wool fibres (polyester 66.5 µm ± 11.1 compared to an average length of 30.4 µm ± 4.4, *p* ≤ 0.016) (Figure 5). There was no significant difference in translocation length between cells on polyester fibres and cells alone in the absence of fungal mycelium or textile fibres (polyester 66.5 ± 11.1 µm compared to no fibre/mycelium 55.4 ± 9.2 µm, *p* ≤ 0.314) (Figure 5). Around the fibres where no highways were observed, silk and wool, the length of cell translocation appeared significantly shorter than translocation length around polyester fibres or with *P. putida* cells alone (silk 27.5 ± 1.4 µm, wool 27.3 ± 1.2 µm, c. f. polyester 66.5 ± 11.1 µm, No fibre/mycelium 55.3 ± 9.2 µm, *p* ≤ 0.011). No significant differences in cell translocation length were apparent between linen, nylon, silk and wool fibres (*p* ≥ 0.277, Figure 5) or between all fibres tested and translocation length on fungal mycelium (*p* ≥ 0.065) (Figure 5).

### 3.5 Design perspective—The *Fibre Highways Collection*

#### 3.5.1 Knit as a scaffold for mycelium growth

**FIGURE 6:**
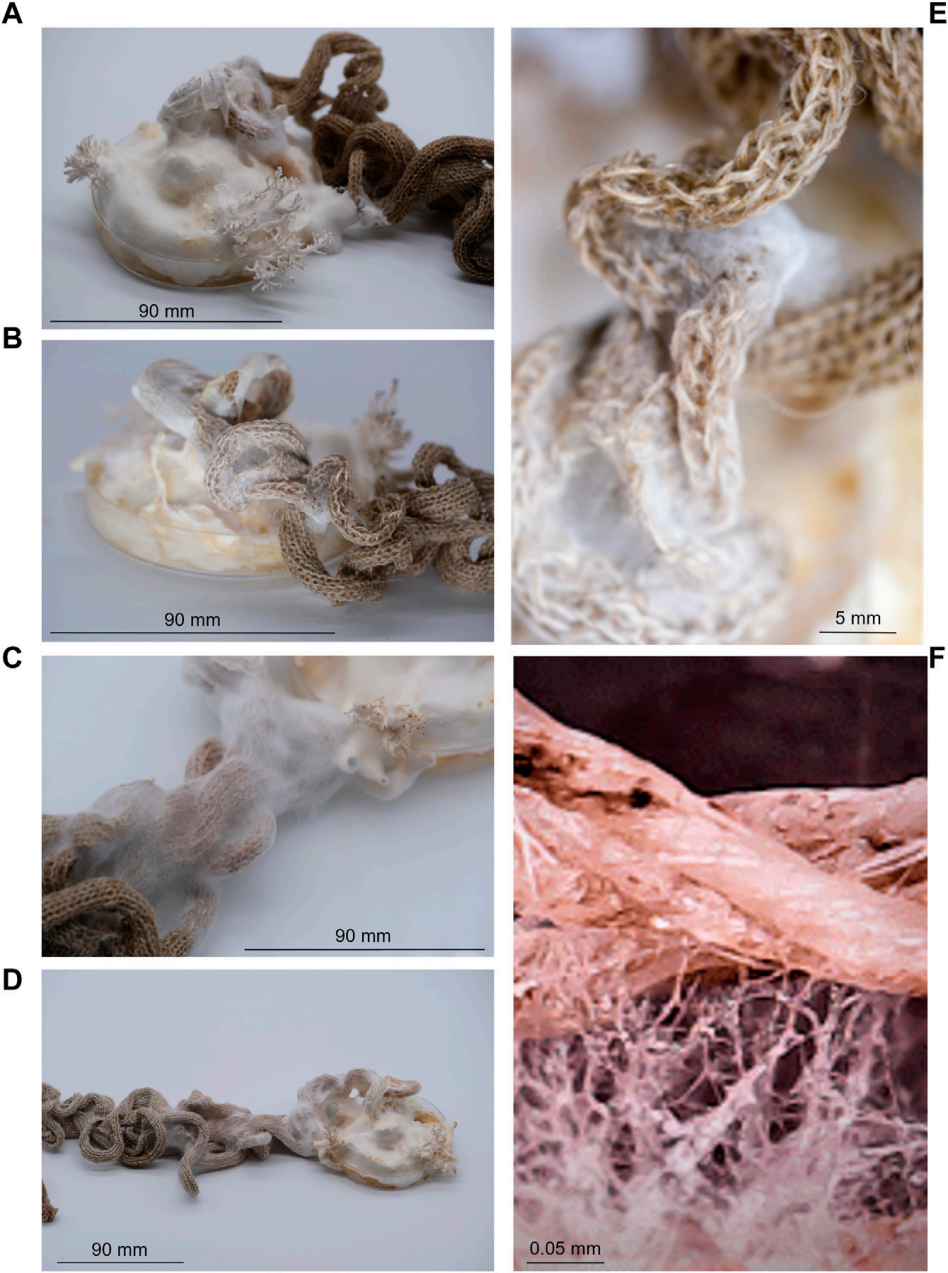
Growth of fungal mycelium (*Pleurotus ostreatus*) through knitted structures over a six-week period, showing growth after 1 week **(A)**, 2 weeks **(B)**, 4 weeks **(C)**, 6 weeks **(D)** and with closer proximity to visualise fibre-mycelium interactions **(E, F)**. Highlighting the ability for natural fabrics to provide a scaffold to support microbial communities.

The fungus, *P. ostreatus*, was grown in association with knitted fabrics to observe if the knit could act as a scaffold, directing the growth of mycelium. Growth was observed along the knitted fabric over the 6-week growing period. Mycelium growth only occurred along the knitted fabric. After 1 week, hyphae were visible on the fabric in contact with the growing mycelium (Figure 6). After 2 weeks, mycelium growth stretched along individual curling sections of fabric. After 6 weeks, the growth extended to 13 cm along the knitted fabric (Figure 6). No growth was observed spreading from the Petri dish in other directions at any point during the six-week period.

#### 3.5.2 Fibre Highways Collection

**FIGURE 7:**
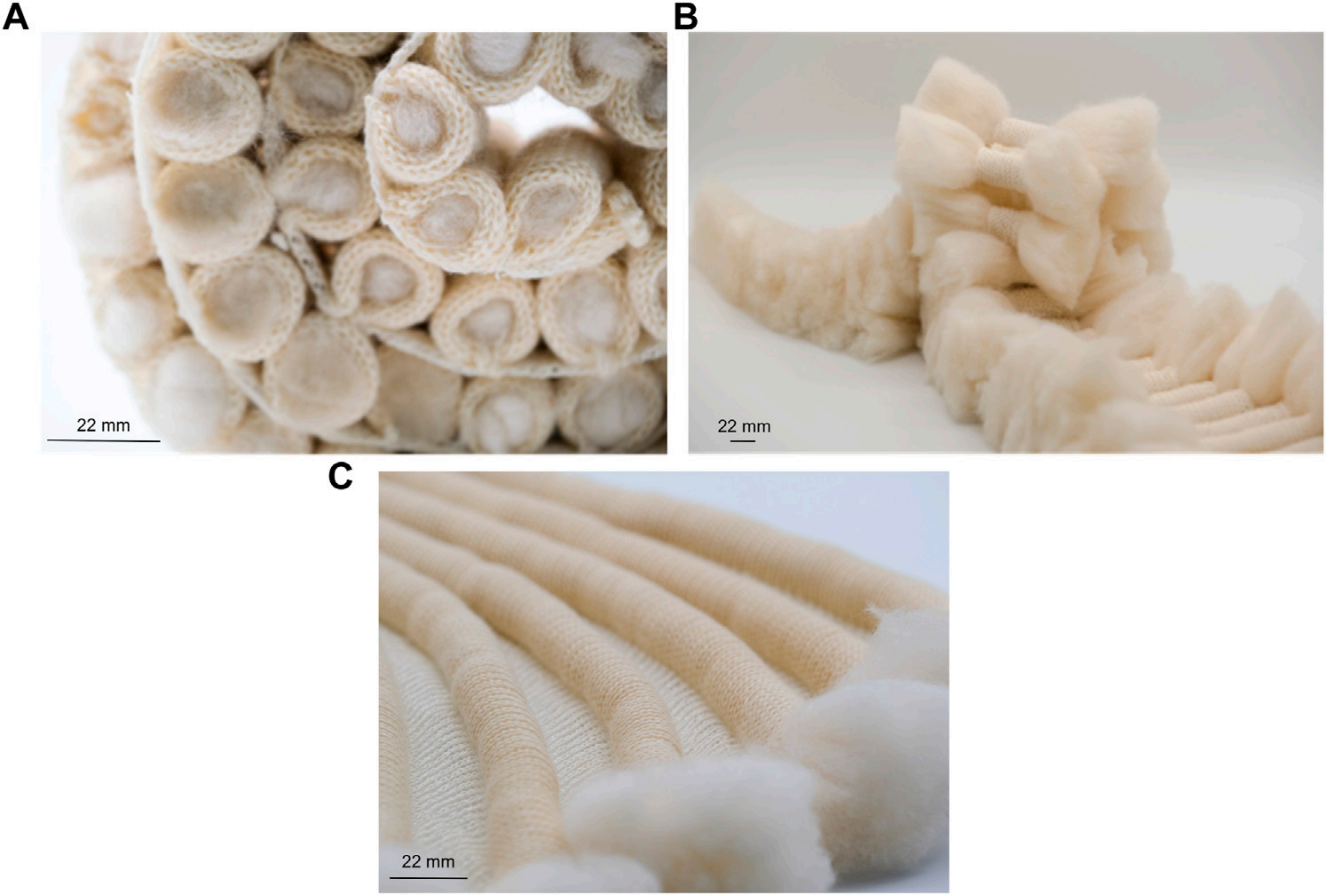
Textiles were engineered to produce shape-changing materials through modified liquid absorption rate [**(A, B)** and Supplementary Video S9] and enhancements in surface area **(B, C)** for adaptation to oil spill capture.

**FIGURE 8:**
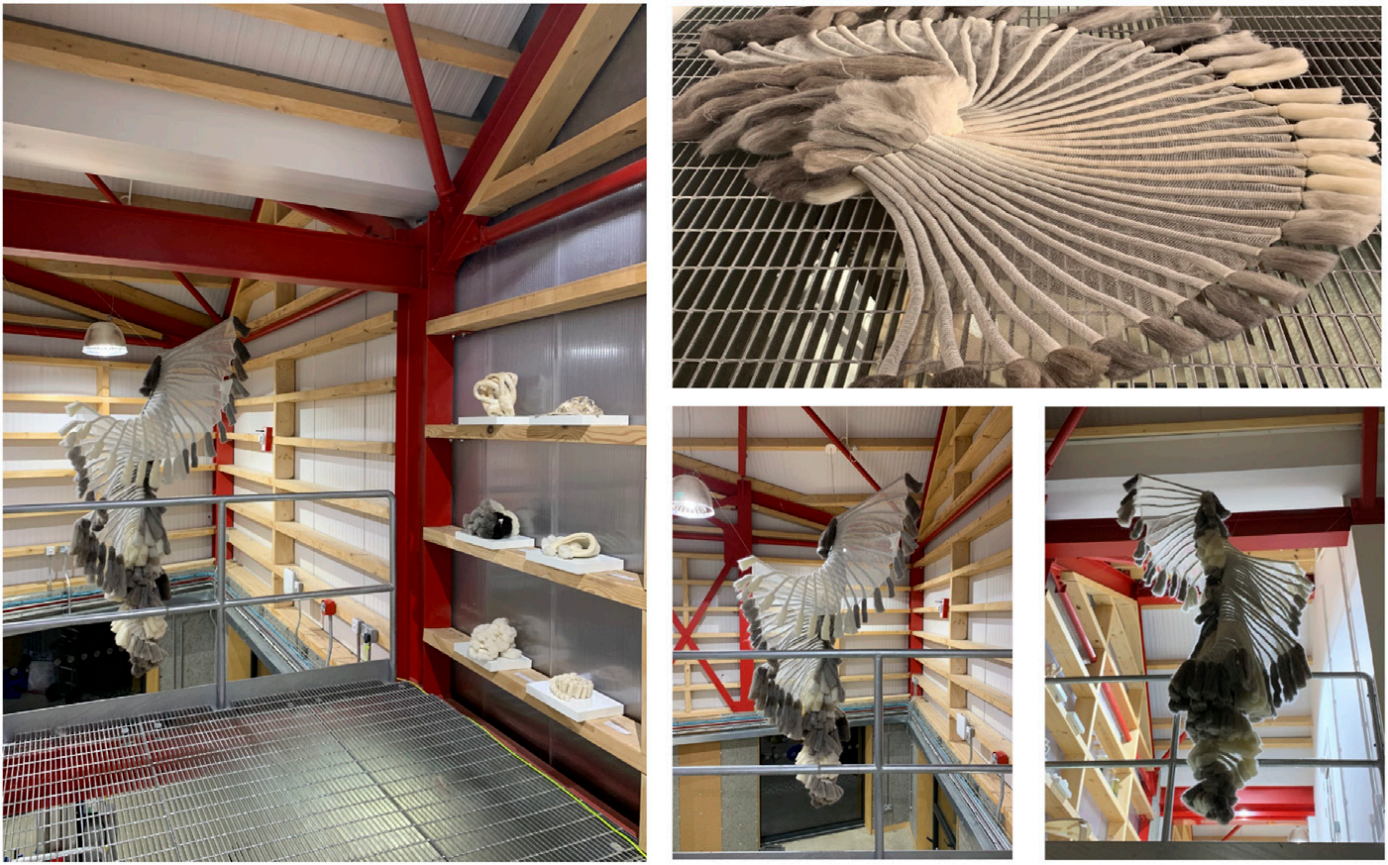
The *Fibre Highways Collection*, the design installation was influenced by confocal microscopy images during the study. The final design piece, Ebb&Flow, transitions through a colour-changing gradient of selected wool fibres conceptualising both the uptake of a hydrocarbon pollutant by fibres and the translocation of microbes along textile fibres to promote oil biodegradation.

The collection comprised liquid-activated shape-changing knitted fabrics. Using the hydrophillic properties of natural fibres, the research explored how to use the different liquid absorption and retention rates of wool and linen to engineer shape-changing knitted fabrics suitable for adaptation to oil spill capture (Figures 7A, B and Supplementary Video S9). Fabrics were actuated using water sprays to demonstrate responsive properties. Enhancements in surface area, and the integration of loose wool fibres within the structures were also designed and explored in the collection for adaptation to oil spill clean-up (Figures 7B, C). In the final exhibition, the *Fibre Highways Collection* demonstrated the potential to scale up the responsive capacities of the material system using locally produced UK wool. The final piece, Ebb&Flow, conceptualised both the uptake of a hydrocarbon pollutant by fibres and the translocation of microbes along fibre highways via transitions through a colour-changing gradient of selected wool fibres (Figure 8).

## 4 Discussion

### 4.1 Translocation of *Pseudomonas putida* on fibre highways

The study demonstrated the proof of concept that motile bacteria can translocate around textile fibres. *Pseudomonas putida* were shown to utilise a water layer surrounding the fibres, supporting the hypothesis that fibres can aid bacterial translocation through their use as ‘fibre highways’. The motile bacterial cells were shown to translocate around a liquid layer surrounding polyester, nylon, and linen fibres, yet no evidence of translocation was apparent on silk or wool fibres, suggesting microbes elicit different responses to specific fibre types (Figures 4, 5, Supplementary materials S1A–C to S7A–C). Digital particle tracking of the cells translocating on an aqueous layer surrounding fungal mycelium and fibres was performed which showed that *P. putida* exhibited movement in both the *x* and *y* direction over a distance of microns, suggesting cell viability and motility rather than the random motion of Brownian movement. Cells translocated the furthest on synthetic polyester fibres (Figure 5), the only fibres tested with hydrophobic properties (Table 1).

Bacterial motility is often driven by chemotaxis—sensing of the chemical environment with movement towards substrates or nutrients and away from adverse, toxic molecules (Wadhams & Armitage, 2004). *Pseudomonas* are known to be chemotactic and have been shown to respond to a wide range of molecules (reviewed in Sampedro et al., 2015). Although it was not the focus of the current study, the translocation of *Pseudomonas* on both fungal and fibre highways is likely to be chemotactically driven. Metabolomic approaches to determine the range of metabolites from fungal exudates during bacterial translocation on fungal highways may provide further insights into the processes of chemotaxis. The aim of metabolomic studies with respect to the research performed herein would assist in determining the role of fungal/bacterial metabolites or fungal exudates which may be pertinent to chemotactic bacterial translocation on fungal or fibre highways, relative to highway-free controls. Rapid micro-scale solvent extraction for screening of fungal pure cultures for metabolites followed by high-performance liquid chromatographic (HPLC) analysis could be used, which was previously used to determine chromatographic metabolite profiles from 395 fungal isolates (Smedsgaard, J. 1997). Alternatively, Orbitrap high resolution mass spectrometry is an untargeted approach for the determination of bacterial or fungal metabolites (Arroyo-Manzanares et al., 2015), which could be used for comparative analyses in samples with and without fungal/fibre highways for the detection and identification of differentially expressed metabolites. With respect to oil bioremediation, metabolomic approaches would facilitate in understanding the dual fungal and bacterial attack on hydrocarbons in response to the application of fungal/fibre highways. This warrants further investigation to elucidate the suite of intracellular and extracellular enzymes and metabolites involved in the complex process of hydrocarbon biodegradation within natural environments (Dombrowski et al., 2016; Li et al., 2020).

### 4.2 Effect of fibre morphology, physicochemical parameters and wicking characteristics on fibre highways

Fibre diameter varied among synthetic and natural fibres. The synthetics, nylon and polyester, showed greater diameter compared to natural linen, wool and silk (Table 1). Among the natural fibres, linen was observed to have the greater diameter (Table 1). Interestingly, longer bacterial translocation length was more readily observed around fibres with greater diameter (polyester, nylon and linen, Figure 5). As reported, surface characteristics may influence microbial attachment (Yuan et al., 2017; Varshney et al., 2021), and therefore are also likely to affect bacterial translocation. Fibre diameter combined with other surface characteristics such as hydrophobicity and electrical charge may have influenced this process.

The morphological structure of the textile fibres (Figure 3) was important to consider in relation to the translocation of microorganisms (Figures 4, 5). It is possible that the cuticle scales on merino wool could interfere with or inhibit translocation of the cells around the fibres (Figure 3E; 4F). Merino wool and its components have recently been assessed for their antibacterial properties (Caven et al., 2019), where raw untreated wool showed no antibacterial properties and appeared as a suitable host for bacterial growth. However, when the wool fibres were broken down into their constituent parts the presence of excess water during antibacterial testing was shown to have a significant impact on the antibacterial properties of the cuticle scales and the cortical cells (Caven et al., 2019). It was suggested that water plays a key role in the wool fibre component’s relationship with bacteria, which aligns with the findings of this study as wool was shown to retain the highest percentage of water in wicking tests (Table 1) and bacterial translocation was not apparent (Figures 4F, 5). By contrast, silk fibres showed the least wicking velocity and percentage (Table 1) and it is therefore possible that with this fibre type a lack of water may have inhibited bacterial translocation (Figures 4E, 5). Interestingly, silk fibroin has previously been shown to provide a substrate for the growth of various bacterial species and biofilms due to surface hydrophobicity, charge, chemical composition and wettability (Ghalei & Handa., 2022), however the mechanisms of interactions of silk and bacterial translocation remain unclear and warrant further investigation.

### 4.3 Design perspective—The *Fibre Highways Collection*

From a design perspective the approach was to develop a multi-kingdom textile ecosystem, using knitting as a tool for conceptual thinking, a microbial habitat, a transport system, and an active agent in remediation. *Fibre Highways* anticipated how a symbiotic relationship could be developed between knitted fabrics and microorganisms, selecting specific textile materials and knitted structures to culture responsive living textiles that supported microbial communities. Knitted fabric provided a unique textile structure to transform the potential of bacterial translocation along fibre highways into responsive hybrid systems for targeted bioremediation. The fabric collection applied the findings of fabric wicking tests in combination with the results of translocation experiments. Wool and linen yarns were selected for fabric design. Wool demonstrated the highest capacity to retain water in comparison to other fibres (Table 1). This provided the mechanism to hold liquid in sections of fabric filled with loose wool fibres. Linen showed the fastest wicking rate of the natural fibres tested (Table 1), this property was used specifically to generate moisture responsive shape change in fabrics. Translocation of *P. putida* was observed on linen fibres but not on wool (Figures 4, 5, Supplementary Videos S2A–C, S6A–C), this was applied so that sections of linen fabric could be in direct contact with external surfaces to position bacterial translocation for oil spill capture (Figure 7). At a micro scale, microbial habitats were integrated through the selection of fibres and yarns suitable for microbial translocation. At a macro scale the textiles themselves were engineered for variable liquid absorption rates and environmentally responsive shape-change using Programmable Knitting. Through a design lens the collaboration moved between the micro and the macro, the bio, the myco and the textile.

The growth of fungal mycelium through knitted structures was demonstrated (Figure 6), highlighting the ability for natural fabrics to support microbial communities whilst retaining the ability to produce environmentally responsive shape-change. The final design piece, Ebb&Flow, incorporated shape-change functionality into a wave of knitted fabric descending the interior of the OME, a unique experimental living house (Figure 8). The form was expressed through integrated shaping, interspersed with rolls of fabric and structured by individual bundles of wool roving. The installation embodied the findings of *Fibre Highways*: fibres and yarns were selected to facilitate variable moisture absorption properties and encourage microbial interactions. Translocation was articulated through the movement of the fabric itself which transitioned through a colour-changing gradient of selected wool fibres reflecting the uptake of a hydrocarbon pollutant. Ebb&Flow was moisture sensitive, translating the concept of bacterial translocation into a macroscale behaving textile that forms and reforms through cycles of saturation (Figure 8).

Components of the *Fibre Highways Collection* were exhibited as “Responsive Textiles” at the Du Sensorial au Biomimétisme (Sensory to Biomimicry) as part of the Saint-Etienne International Design Biennale (April—July 2022). Knitted fabrics designed to promote and disseminate microbial:textile interactions were showcased.

### 4.4 Areas of Application and future Direction

The application of this particular study centred around the future use of biohybrids as a biotechnology to improve oil bioremediation by transferring active hydrocarbon-degrading microbes into polluted environments via fungal/fibre highways. Preliminary findings of this study showed that bacterial translocation activity around fungal highways did not diminish in the presence of crude oil, known to contain an abundance of toxic compounds, in comparison to the oil-free controls. A deeper understanding of the movement and interactions of microbes on fungal mycelium and on natural and synthetic fibres will likely lead to the development of biohybrid textiles that can be used to, for example.

• increase the contact time of microbes with the pollutant for more efficient bioremediation
• seed polluted sites which may be difficult to reach with fibre highways e.g. fat, oil and grease in sewer systems
• provide a dual attack on hydrocarbons by both fungi and bacteria supported by environmentally responsive textiles

Future studies would incorporate monitoring of the degradation of hydrocarbon pollutants over time in ecosystems exposed to fungal and/or fibre highways. To assess the degree of hydrocarbon degradation analytical oil geochemistry methods would be deployed, including solid phase extraction to retrieve the total hydrocarbon fraction, followed by gas-chromatography-flame ionisation detection (GC-FID) to determine degradation of the most labile *n*-alkane fraction of the crude oil (Sherry et al., 2020), relative to oil-free and highway-free controls. The development of highly functionalised, sustainable, smart materials for pollutant degradation at the intersection of microbiology, biodesign, and textiles, will assist in reconsidering the way in which hydrocarbon contaminated sites are currently bioremediated, optimising existing treatment processes to create more sustainable biotechnologies and significantly reducing environmental pollution. There remains huge scope to further explore the relationships between environmental microbiology and textile design and technology to enable textiles to become an active material component that could be pertinent in pollutant bioremediation.

## Data Availability

The original contributions presented in the study are included in the article/Supplementary Material, further inquiries can be directed to the corresponding author.
