# Supplementary material for: Biohybrids: Textile fibres provide scaffolds and highways for microbial translocation
Source: Front Bioeng Biotechnol. 2023 Jun 13;11:1188965. doi: 10.3389/fbioe.2023.1188965 (PMC10293675; doi:10.3389/fbioe.2023.1188965)
Supplement: Supplementary file 2 [file Table1.docx]

Supplementary Material

Image attributions for Figure 1 from bioicons (https://bioicons.com):

Confocal Laser Scanning Microscope icon by DBCLS (https://togotv.dbcls.jp/en/pics.html) is licensed under CC-BY 4.0 Unported (https://creativecommons.org/licenses/by/4.0/)

Petri-dish-lid-yellow icon is by Servier (https://smart.servier.com/) is licensed under CC-BY 3.0 Unported (https://creativecommons.org/licenses/by/3.0/). The original work has been modified: window added to indicate window within agar and fungal mycelium drawn in corners of agar window.

Micropipette icon is by Servier (https://smart.servier.com/) licensed under CC-BY 3.0 Unported (https://creativecommons.org/licenses/by/3.0/). The original work has been modified: tip coloured beige to indicate bacterial culture inside micropipette tip.

Camera icon by OpenClipart (https://openclipart.org/) is licensed under CC0 (<https://creativecommons.org/publicdomain/zero/1.0/>)

Single bed knitting machine (Silver Reed, UK) (<https://silverviscount.co.uk/index.php?route=product/product&product_id=18&limit=10>)

*Pseudomonas putida* on *Cunninghamella elegans* - Fungal highway (3 replicates):

Video 1a: Series006

Video 1b: Series032

Video 1c: Series034

Video 1d: Series021_Oil (in presence of crude oil)

Supplementary material Video 1a-d. Time-lapse confocal microscopy showing motile bacteria, *Pseudomonas putida*, utilising a water layer surrounding fungal mycelium (*Cunninghamella elegans*) for translocation, termed 'fungal highways' (recreated from <https://youtu.be/AnsYh6511Ic> (Berthold & Wiedling, 2016)).

*Pseudomonas putida* on Linen fibres:

Video 2a: Series025

Video 2b: Series027

Video 2c: Series040

*Pseudomonas putida* on Nylon fibres:

Video 3a: Series051

Video 3b: Series064

Video 3c: Series067

*Pseudomonas putida* on Polyester fibres:

Video 4a: Series085

Video 4b: Series087

Video 4c: Series091

*Pseudomonas putida* on Silk fibres:

Video 5a: Series050

Video 5b: Series060

Video 5c: Series072

*Pseudomonas putida* on Merino wool fibres:

Video 6a: Series013

Video 6b: Series023

Video 6c: Series039

*Pseudomonas putida* control (no fungal mycelium or fibres):

Video 7a: PseudoControlR1

Video 7b: PseudoControlR2

Video 7c: PseudoControlR3

Supplementary material Video 2-7 a-c. Time-lapse confocal microscopy showing motile bacteria, *Pseudomonas putida*, utilising a water layer surrounding textile fibres for translocation, termed 'fibre highways’: Linen (Video 2a-c), Nylon (Video 3a-c), Polyester (Video 4a-c), Silk (Video 5a-c) and Wool (Video 6a-c). *Pseudomonas putida* control without fungal or fibre present as comparator (Video 7a-c).

Video 8a: Movie PseudoControlR2.

Video 8b: Movie Series032.

Video 8c: Movie Series025.

Supplementary material Video 8 a-c. Examples of particle tracking of *Pseudomonas putida* cells translocating alone (a), on *C. elegans* fungal mycelium (b) or on linen fibre (c).

Video 9

Supplementary material Video 9. Time-lapse of moisture responsive knitted textile.
